# Fatal Coxsackievirus A-16 Pneumonitis in Adult

**DOI:** 10.3201/eid1307.070295

**Published:** 2007-07

**Authors:** François Legay, Nicolas Lévêque, Arnaud Gacouin, Pierre Tattevin, Julien Bouet, Rémi Thomas, Jean-Jacques Chomel

**Affiliations:** * Centre Hospitalier Universitaire Pontchaillou, Rennes, France; † Centre Hospitalier Universitaire Edouard Herriot, Lyon, France; 1 Current affiliation: Centre Hospitalier Saint-Brieuc, Saint-Brieuc, France

**Keywords:** pneumonitis, hand, foot, and mouth disease, enterovirus, coxsackievirus A-16, dispatch

## Abstract

Coxsackievirus A-16 (CVA-16) is the agent of hand, foot, and mouth disease in children. We report a case of fatal pneumonitis in an adult due to a CVA-16 strain with a low (78.6%) rate of sequence homology with the reference strain. A modified, more virulent, strain of CVA-16 could be emerging.

Hand, foot, and mouth disease (HFMD) is a benign condition caused by coxsackievirus A type 16 (CVA-16), which affects young children and usually resolves uneventfully. Rarely, it may be associated with complications such as meningitis, encephalitis, myelitis, and respiratory failure. During outbreaks of enterovirus infection, respiratory failure has been associated with cardiac failure in children infected with enterovirus 71 (EV 71) but not in those infected with CVA-16 (*1*,*2*). We report a case of CVA-16 pneumonitis that was fatal for an adult.

## The Case

In April 2006, a 76-year-old man was admitted to the emergency department of Pontchaillou University Hospital, Rennes, France, with acute onset of fever, lumbar pain, and dyspnea. Examination found a temperature of 37.9°C and bilateral pulmonary crackles. Laboratory results were the following: leukocytes 9,600 cells ×10^6^/L (90.6% neutrophils), C-reactive protein 216 mg/L (normal value <5 mg/L), and arterial oxygen partial pressure 67 mm Hg (room air). Chest radiograph was unremarkable, and *Legionella pneumophila* urinary antigen was not found. The patient was treated with amoxicillin-clavulanate and ofloxacin. On day 3, acute respiratory distress syndrome developed, and the patient required mechanical ventilation. Computed tomographic scan of the thorax showed bilateral alveolo-interstitial infiltrates (Figure). Transthoracic echocardiograph and pulmonary artery catheterization showed normal left ventricular function. Serum troponin levels were within normal limits.

**Figure:**
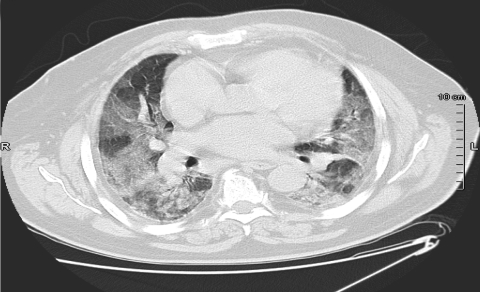
Thoracic computed tomographic scan on day 3, showing bilateral alveolo-interstitial infiltrates.

A bronchoalveolar lavage (BAL) was performed; no pyogenic bacteria, *L. pneumophila*, *Mycobacterium tuberculosis*, *Pneumocystis jiroveci,* or *Aspergillus* sp. were isolated despite appropriate staining for direct examination and cultures on appropriate media. Negative results were obtained in *M. tuberculosis* PCR and immunofluorescence assays (IFAs) for *P. jiroveci* and *L. pneumophila*. The result of a PCR for human herpesviruses, which used herpes consensus identification, was negative. Results of testing for respiratory syncytial virus, influenza virus, parainfluenza virus, and adenovirus by IFA and ELISA, which used specific monoclonal antibody, were all negative. In addition, serologic test results for *L. pneumophila*, *Mycoplasma pneumoniae*, *Chlamydophila pneumoniae*, *C.* *psittaci*, *Coxiella burnetti,* and HIV were negative. Three sets of blood cultures taken at the time of hospital admission remained sterile. Test results for antinuclear factors, antiglomerular basement-membrane antibodies, and antineutrophil cytoplasmic antibodies were negative.

BAL was positive in 2 enteroviral PCR assays that used EV1 primer from Rotbart et al. (*3*) and real-time PCR with primers and probe adapted from Verstrepen et al. (*4*). In addition, on day 3, MRC-5 cell culture showed a specific cytopathic effect, which was confirmed as enterovirus by indirect IFA that used enterovirus mouse monoclonal antibody (Novocastra, Newcastle, UK) and fluorescein isothiocyanate–conjugated AffiniPure Goat Anti-Mouse (Jackson Immuno-Research, West Grove, PA, USA). Enterovirus was also detected in serum and pharyngeal samples by real-time PCR. Results of real-time PCR and cell culture (MRC-5 and LLC-MK2) were also positive for enterovirus on BAL performed on days 8 and 14. Kinetics analysis in real-time PCR showed a 100-fold decrease in viral load by comparison of cycle thresholds between day 8 and day 14 BAL. Serologic testing for enterovirus showed an 8-fold increase in enterovirus antibody titration by complement fixation between days 3 and 11. Immunoglobulin M to echovirus/coxsackievirus was detected by ELISA in serum on day 11 (Genzyme, Virotech, Chilly Mazarin, France).

No drug is currently approved for the treatment of enterovirus infection. Pleconaril may be of value in severe enteroviral infections (*5*) but is no longer available because licensure was not pursued. The patient did not improve and died on day 28 of intractable hypoxemia. Histologic examination of postmortem pulmonary biopsy specimen showed diffuse alveolar damage and fibrosis, real-time PCR detected enterovirus, and viral cultures were negative. The enterovirus isolated on the day-3 BAL was identified as CVA-16 by partial sequencing of the VP1 region that encompasses the BC loop. This region was amplified with primers 292 as sense primer and 222 as antisense primer (*6*). The sequences obtained (reference no. bankit 845096-DQ993173 until definitive number assigned) were aligned with the corresponding region in GenBank. Comparison of a 338-nt sequence with that of the CVA-16 reference strain (prototype BrCr) and the EV 71 reference strain (prototype G-10) showed nucleotide identity rates of 78.6% and 64.6%, respectively.

On subsequent questioning, the patient’s wife reported that he had had close contact with his granddaughter, who had active HFMD, while in the United Kingdom during the week before he was admitted. Although a large HFMD outbreak occurred in the United Kingdom in 1994 (*7*), no enterovirus outbreaks had been reported at the time of the patient’s admission. During the 1994 UK outbreak, CVA was isolated in 28 of 40 patients (type CVA-16 in 21 patients and CVA-10 in 7 patients), but secondary cases in family members were rare and no case of pneumonitis or death was reported.

## Conclusions

Fatal CVA-16 infection has been described infrequently in children who had HFMD associated with myocarditis (*8*). We report a fatal CVA-16 infection associated with pneumonitis in an adult; to our knowledge, this is the first such report. Our patient had neither myocarditis nor left ventricular dysfunction, as demonstrated by pulmonary artery catheterization results, echocardiograph results, and serum troponin levels. In 2003, 7 fatal cases of HFMD in children were reported in Singapore (*9*). These children had interstitial pneumonitis, either alone or associated with myocarditis or encephalitis. EV 71 was isolated in 4 cases and echovirus 16 in 1. The CVA-16 strain isolated from our patient had a low percentage of nucleotide identity with the reference strain (78.6%); a threshold of 90% is usually required to define strain homology. This may be a sign that this virus is evolving. A strain similar to that from our patient was circulating in China from 1999 through 2004 (98% nucleotide identity; GenBank accession no. AY821798) and was isolated from fecal samples of children with HFMD or suspected enterovirus infection (*10*). This strain was associated with local yearly outbreaks in which only a few cases of neurologic disease and no deaths were reported. According to phylogenetic analysis based on VP4 207-bp nucleotide sequence, the authors concluded that 3 genetic lineages were circulating in Asia at that time and suggested that the same tendency may apply in other continents (*10*).

We report what we believe to be the first case of CVA-16 pneumonitis in an adult, with fatal outcome. Preliminary sequence analysis revealed a low rate of homology between the CVA-16 strain we isolated and those previously published, which suggests that a new, more virulent, strain of CVA-16 could be emerging. To compare the sequences to those published in GenBank, we are sequencing the complete part of the genome encoding the VP1 capsid protein.
